# Spermine Confers Stress Resilience by Modulating Abscisic Acid Biosynthesis and Stress Responses in Arabidopsis Plants

**DOI:** 10.3389/fpls.2019.00972

**Published:** 2019-07-31

**Authors:** Francisco Marco, Enrique Busó, Teresa Lafuente, Pedro Carrasco

**Affiliations:** ^1^Estructura de Recerca Interdisciplinar en Biotecnologia i Biomedicina (ERI BIOTECMED), Universitat de València, Valencia, Spain; ^2^UCIM, Universitat de València, Valencia, Spain; ^3^Departamento de Biotecnologia de Alimentos, Instituto de Agroquímica y Tecnología de Alimentos, CSIC, Valencia, Spain

**Keywords:** abscisic acid, spermine, salt stress, stress response, stress tolerance

## Abstract

Polyamines (PAs) constitute a group of low molecular weight aliphatic amines that have been implicated as key players in growth and development processes, as well as in the response to biotic and abiotic stresses. Transgenic plants overexpressing PA-biosynthetic genes show increased tolerance to abiotic stress. Therein, abscisic acid (ABA) is the hormone involved in plant responses to environmental stresses such as drought or high salinity. An increase in the level of free spermine (Spm) in transgenic Arabidopsis plants resulted in increased levels of endogenous ABA and promoted, in a Spm-dependent way, transcription of different ABA inducible genes. This phenotype was only partially reversed by blocking ABA biosynthesis, indicating an ABA independent response mediated by Spm. Moreover, the phenotype was reproduced by adding Spm to Col0 wild-type Arabidopsis plants. In contrast, Spm-deficient mutants showed a lower tolerance to salt stress. These results indicate that Spm plays a key role in modulating plant stress responses.

## Introduction

Polyamines (PAs) are small aliphatic amines found in all living organisms (Kusano et al., 2008). In plants, the three major PAs include putrescine (Put), spermidine (Spd), and spermine (Spm). In Arabidopsis, Put biosynthesis is done mainly by arginine decarboxylase (ADC; EC4.1.1.19) activity, which decarboxylates arginine as the first step in the PA-biosynthetic pathway. Put serves as the precursor for higher molecular weight PAs Spd and Spm. Spd synthase (SPDS;EC2.5.1.16) and Spm synthase (SPMS;EC 2.5.1.22), respectively, catalyze the addition to Put of aminopropyl groups generated from S-adenosylmethionine (SAM) by SAM decarboxylase (SAMDC, EC 4.1.1.50) (Tiburcio et al., 1990). Arabidopsis genome carries two genes that encode for ADC enzyme (ADC1 and ADC2; Urano et al., 2005), four genes for SAMDC (SAMDC1-4; Urano et al., 2003), two genes for SPDS (*SPDS1* and *SPDS2*; Hanzawa et al., 2002) and one single gene for SPMS (Hanzawa et al., 2002). *ACAULIS5* (*ACL5* gene), originally assigned as a putative Spm synthase (Hanzawa et al., 2000), encodes a thermospermine (tSpm) synthase (Knott et al., 2007). Free PAs levels of the plant are the result of a balance between biosynthesis and degradation, the former process done mainly through the activity of diamine oxidases (DAO, EC1.4.3.6) and polyamine oxidases (PAO, EC1.5.3.3), that exhibit different substrate preferences. Diamines like Put are a preferred substrate for DAOs, while higher molecular weight PAs, like Spm, tSpm, and Spd, are oxidized by PAOs (Cona et al., 2006). Some PAOs terminally oxidize PAs, while other isoforms are involved in a PA back-conversion process of tSpm to Spm and Put, releasing H_2_O_2_ (Moschou et al., 2012). Ten genes encoding DAOs, as well as five genes that encode PAOs are present in the Arabidopsis genome (Takahashi et al., 2010; Planas-Portell et al., 2013).

As sessile organisms, plants respond to environmental stresses through a series of physiological, cellular, and molecular changes. Consequence of these changes, transcriptomic, proteomic, and metabolic modifications, which help to mitigate the effect of stress and lead to the adaptation of the plant, occur. Classically, PAs have been assigned to play an important role in modulating the response of plants to diverse environmental stresses (Bouchereau et al., 1999). Elevated PA levels are one of the most remarkable metabolic hallmarks in plants exposed to stresses such as drought, salinity, chilling, heat, hypoxia, ozone, UV, or heavy metals (Alcázar et al., 2010; Gill and Tuteja, 2010). In the last years, it has been elegantly summarized the relationship between the functional significance of PAs and their roles in tolerance and/or amelioration of stress responses in plants (Minocha et al., 2014). It has been suggested that PAs could act as stress messengers in plant responses interacting with different stress pathways (Takahashi et al., 2003; Tuteja and Sopory, 2008; Alcázar et al., 2010; Marco et al., 2011).

It is well known that abscisic acid (ABA) is involved in the response of plants to abiotic stresses (Vishwakarma et al., 2017). Abscisic acid (ABA) is a plant hormone that quickly accumulates in plants exposed to different abiotic stresses. ABA is a sesquiterpenoid that is synthetized from C40 carotenoids in three steps catalyzed by zeaxanthin epoxidase (Marin et al., 1996), 9-cis-epoxycarotenoid dioxygenase (NCED; Schwartz et al., 1997) and abscisic aldehyde oxidase (AAO; Seo et al., 2000). The key step in ABA biosynthesis is the cleavage reaction of epoxy carotenoids to produce xanthoxin catalyzed by NCED (Kende and Zeevaart, 1997). In fact, ectopic expression of NCED causes overproduction of ABA in tobacco and tomato (Thompson et al., 2000). Accumulation of ABA stimulates stomatal closure to limit water loss from leaves, as well as changes in the level of expression of genes involved in stress responses and in the biosynthesis of osmoprotectant species that help the cells to handle damage and restore cellular homeostasis (Fujita et al., 2011). Transcriptome comparison of Arabidopsis plants exposed to salt or drought stress shows that about half of the genes induced by these stresses are also induced by ABA (Seki et al., 2002). Moreover, the disruption of ABA synthesis by transgenic approaches in Arabidopsis leads to plants more sensitive to drought stress (Iuchi et al., 2001). These observations suggest the predominant role of this hormone as a signaling molecule in the response against drought and high salinity stresses (Sah et al., 2016).

Hierarchical clustering expression analyses in Arabidopsis using public microarray data indicate that some, but not all, PA biosynthesis gene paralogs share similar expression patterns, in agreement with their different implications in stress and development (Tiburcio et al., 2014). Moreover, transcriptome studies performed in Arabidopsis have revealed differential regulation of PA biosynthesis genes by abiotic stress (Alcazar et al., 2006b). Recent studies have been reported that Spm-overproducer plants overexpress a number of ABA related genes (Marco et al., 2011). On the other hand, the characterization of PA loss-of-function mutants has provided evidence for the involvement of PAs in resistance traits (Urano et al., 2003; Yamaguchi et al., 2007; Cuevas et al., 2008; Zarza et al., 2017). Besides that, although taken together these evidences suggest that PAs are involved in modulating plant responses to abiotic stress, the relationship between PA, and ABA remains to be fully understood.

In this work, we have observed that overexpression of *SAMDC1* gene in Arabidopsis produces Spm accumulation and leads to plants with an improved tolerance to salt stress. Furthermore, since overexpression of the *NCED3* gene and ABA accumulation were observed on these Spm-accumulating transgenic lines, the expression of several ABA-response related genes was checked in plants with different Spm levels, several Spm-deficient mutant lines, as well as WT plants treated with external Spm. A Spm-dependent expression pattern was observed for *NCDE3* and some ABA-responsive genes. These results suggest that Spm plays a key role in modulating ABA levels and that stress tolerance could be improved by manipulation of Spm biosynthesis, causing the accumulation of endogenous ABA and triggering the expression of a number of stress-response genes. On the other hand, expression of *NCDE3* and other ABA-responsive genes was increased in these Spm-overproducer plants even when ABA biosynthesis was inhibited, suggesting the existence of a Spm-dependent pathway response that would include some ABA-dependent factors.

## Materials and Methods

### Plant Growth Conditions

Experiments were performed using several *Arabidopsis thaliana* (Arabidopsis) lines. Ecotype Col-0, obtained from the Nottingham Arabidopsis Stock Centre (University of Nottingham, Loughborough, UK) was used as the wild type (WT). On the other hand, different lines of Arabidopsis plants with altered Spm levels have been used: three transgenic lines overexpressing the SAMDC1 gene under the control of CaMV35S constitutive promoter (pBISDCs-S3’, pBISDCs-S9’, pBISDCs-S15, Marco et al., 2011), a T-DNA insertion mutant line of the *SPMS* gene (*spms-1*, Imai et al., 2004), an ethyl methane-sulfonate-mutant line of the *ACL5* gene (*acl5-1*, Hanzawa et al., 1997) and a double mutant line obtained from crossing of the two lines mentioned above (*acl5-1/spms-1*, Imai et al., 2004). Both *acl5-1* and *acl5-1/spms-1* mutants showed stem-reduced growth phenotypes (Hanzawa et al., 1997; Imai et al., 2004), however, *spms-1* plants exhibited similar phenotypes to WT plants in terms of growth and development (Imai et al., 2004).

Plants were cultivated in growth chambers Sanyo MLR-350 (Sanyo Electric Co., Japan) under long day conditions, illumination at 23°C for 16 h, in darkness at 16° for 8 h. Previously, all the seeds were stratified for 2 days at 4°C. Adult plants were grown from seeds sown in pots with a 1:1:1 mixture of soil, vermiculite, and sand, and watered with mild nutrient solution [recipe from Arabidopsis Biological Resource Center (ABRC, The Ohio State University, USA) handling plants and seeds guide, http://www.biosci.ohio-state.edu/pcmb/Facilities/abrc/handling.htm].

Five day-old seedlings were grown under the same light and temperature conditions. Seeds were surface sterilized by washing for 10 min in 30% (v/v) commercial bleach, 0.01% (v/v) Triton X-100 and rinsed three times with sterile distilled water. Sterile seeds were plated on 4% agar plates containing one half strength MS medium (Murashige and Skoog, 1962).

### Plant Treatments

Adult plants were grown in pots, salt stress treatments were performed by adding NaCl (0–250 mM) to the watering solution. Visible damage of stress was estimated in 3-week old plants exposed for 10 days to 0–250 mM NaCl, and pictures were taken. Additionally, 4-week old plants were exposed for 6 h to 250 mM NaCl and rosette leaf samples were harvested to isolate RNA. Furthermore, a stress recovery assay was performed by exposing 2-week old plants to 250 mM NaCl for 2 days. After 12 days of recovery in control conditions, stem length and shoot fresh weight (FW) were measured.

Salt stress and inhibition of ABA biosynthesis experiments were also performed in plates by adding 250 mM NaCl to 5-day grown seedlings grown in ½ MS medium with or without 100 μM sodium tungstate dehydrate (Fluka). Entire seedling samples were taken after 6 h.

Seedlings were also grown in ½ MS plates supplemented with 0.1, 0.5, and 1 mM, Put, Spd, or Spm. Entire seedling samples were taken after 5 days of growth. All tissues were harvested and immediately frozen in liquid nitrogen and stored at −80°C until used.

Germination of seeds was also studied in ½ MS plates supplemented with 0, 50, 100, 150, or 250 mM NaCl. Fifty seeds were sowed per plate and the number of seeds that developed cotyledons until 8 days after stratification was counted. All experiments were done by triplicate.

### Quantitative RT-PCR

Total RNA was extracted from plant tissue using Total Quick RNA Cells and Tissues Kit (Talent SRL, Italy), following protocol established by manufacturer. RNA was quantified by their absorbance al 260 nM, and its integrity checked by denaturing agarose gel electrophoresis.

RNA was treated with RNAse free-DNAse (Roche diagnostics, Spain), according to manufacturer’s instructions. A total of 1 μg of DNA free-total RNA was reverse transcribed to First-strand complementary DNA (cDNA) with random hexamers using SuperScript^®^ III First-Strand Synthesis System 1st (Invitrogen, Spain) according to manufacturer’s instructions. Quantitative real time PCR (qRT-PCR) was performed on Gene Amp^R^ 5,700 Sequence Detection System (PE Applied Biosystems, Japan), using Power SYBR^®^ Green PCR Master Mix (PE Applied Biosystems) first-strand cDNA as a template. Each 20 μl reaction contained 1 μl of cDNA, 100 nM of each pair of target primers (FW and REV), and 10 μl of SYBR Green PCR Master Mix. The PCR conditions were as follows: 95°C for 10 min, followed by 40 cycles of 95°C for 30 s and 60°C for 1 min. Three replications were performed for each sample in each experiment. Primers used for real-time PCR are described in Table 1. Data was analyzed according to 2^−ΔΔ*C*T^ Method (Livak and Schmittgen, 2001). Actin-2 (AT5G09810; An et al., 1996) was used as a reference gene.

**Table 1:** Primers used in qRT-PCR analyses.

Gene	AGI locus	Forward (5′–3′)	Reverse (5′–3′)
*COR15A*	AT2G42540	*TGTTCTCACTGGTATGGCTTCTTCT*	*TCTGACAGCGCCGAAGCT*
*NCED3*	AT3G14440	*TCGTCGTACCTGACCAGCAA*	*GACCCACCGGGGATCA*
*RD22BP1*	AT1G32640	*GGTTTCCGGGTCAGATCAATT*	*ATCCCAAACACTCCTCCTTGCT*
*RD26*	AT4G27410	*ACGGTGGTTACGATGCGTTT*	*CCGATTCACATGCCCACTCT*
*RD29A*	AT5G52310	*TGTGCCGACGGGATTTG*	*CTGATGCCTCACCGTATCCA*
*SOS1*	AT2G01980	*AAGGCATTCTCGACAGTGATA*	*TGGATGTAAACGTTATAGCAGA*
*SOS3*	AT5G24270	*CGAAATGGAGTGATCGAGTTTG*	*GCGCGCTTGGATGGAA*
*ACT-2*	AT5G09810	*GATTCAGATGCCCAGAAAGTCTTG*	*TGGATTCCAGCAGCTTCCAT*

### Abscisic Acid Quantification

ABA was measured in seedlings and 4-week old Arabidopsis plants. Plant tissue (0.2 g FW) was homogenized with 5 ml of extraction buffer (80% acetone, 100 mg L^−1^ butylated hydroxytoluene, 0.5 g L^−1^ citric acid) and centrifuged 12,000 ×*g* for 5 min. Supernatant was recovered, dried, and resuspended in 0.5 ml TBS buffer (6.05 g L^−1^ Tris, 0.20 mg L^−1^ MgCl_2_, and 8.8 g L^−1^ NaCl, pH 7.8). ABA was quantified following an indirect ELISA method (Walker-Simmons, 1987). ABA-BSA conjugates were made according to Weiler (1980) as modified by Norman et al. (1988). ABA levels are expressed as ng (g FW)^−1^.

### Statistical Analyses

Data was analyzed by one-way Analysis of Variance (ANOVA) followed by *post-hoc* comparisons by Tukey’s HSD *t* test. A probability level < 0.05 was considered statistically significant. Calculations were performed using IBM^®^ SPSS^®^ Statistics v22.0 Software.

## Results

### pBISDCs Plants Are Tolerant to Salt Stress

In a previous work, we were able to describe that overexpression of SAMDC1 gene in Arabidopsis leads to with higher Spm levels than WT plants (Supplementary Figure S1). Also, the transcriptome of pBISDCs transgenic lines showed an increase in the expression of a set of genes enriched in functional categories involved in defense-related processes against both biotic and abiotic stresses (Marco et al., 2011).

**Figure 1:**
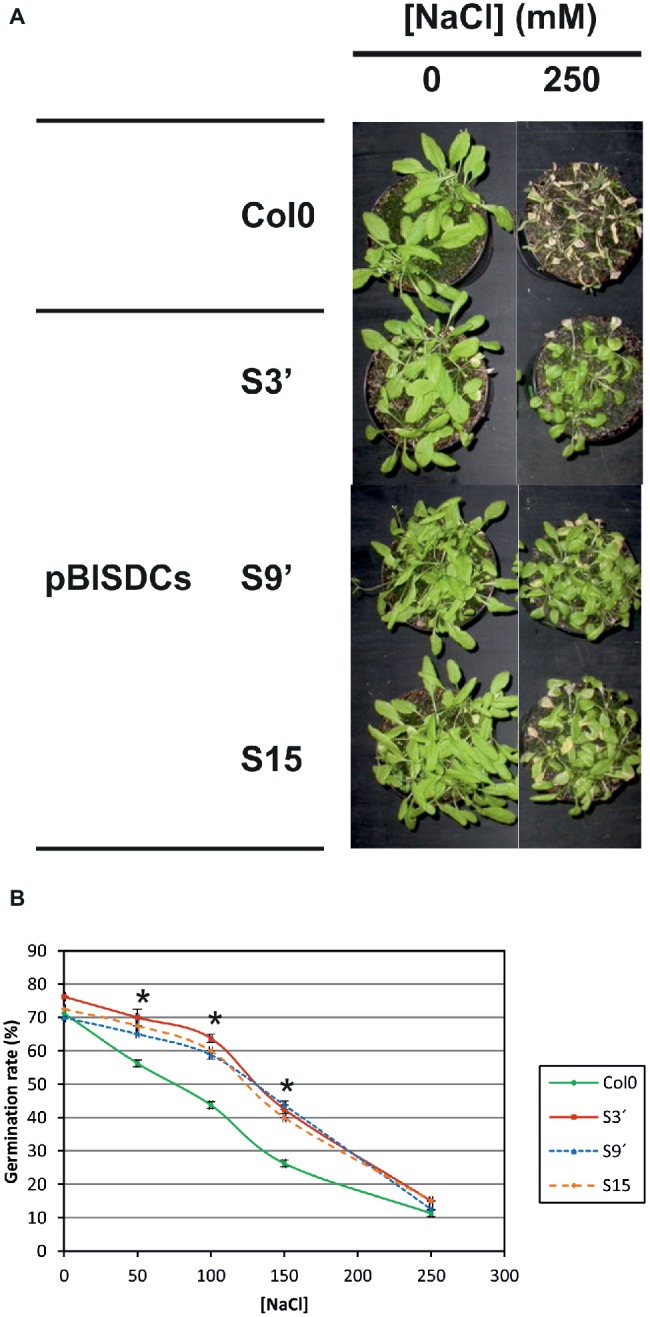
Salt stress tolerance of pBISDCs lines. **(A)** Appearance of Arabidopsis WT and pBISDCs transgenic plants overexpressing SAMDC1 (S3′, S9 and S15) after 10 days of salt treatment. 3 week-old plants were watered with solution without (control) and with the supplementation of 250 mM NaCl. Pictures were taken after 10 days. **(B)** Seed germination percentage in MS plates supplemented with different concentrations of NaCl. The number of seeds that developed cotyledons until 8 days after stratification was counted. Graphs show the mean ± standard deviation from MS plate triplicates. Significant changes from control treatment are highlighted (*) (ANOVA, Tukey HSD test, *p* < 0.05).

To assess the effects of this defense-related transcriptome expression changes in salt stress tolerance, pBISDCs and WT plants were grown in soil for 3 weeks and treated with irrigating solution with presence or absence of NaCl. Under control conditions, pBISDCs showed no phenotype in terms of growth and development. However, after 10 days of treatment, WT plants irrigated with excess NaCl showed generalized chlorosis and wilt, while pBISDCs lines appeared with a healthier aspect and diminished symptoms (Figure 1A; Supplementary Figure S3). A saline stress recovery assay was also performed by exposing pBISDCs and WT plants to 250 mM NaCl during 2 days and leaving them to recover for 12 days in the absence of salt. After the recovery period, salt treated plants showed a reduction in their growth compared to untreated plants, but pBISDCs lines were able to develop higher recovery percentages of stem length (46.1–48.7%) and shoot fresh weight (72.8–75.2%) than WT plants (26 and 54%, respectively) (Supplementary Figure S4).

Additionally, salt stress tolerance was estimated by establishing seed germination percentage after 8 days, of seeds sowed in plates with increasing concentrations of NaCl (0–250 mM). Germination percentage decreased with increasing NaCl concentration for all lines studied (transgenic and WT), dropping to 10% for the highest salt concentration (250 mM) (Figure 1B). However, Spm-accumulating lines maintained better germination percentages (about 15% higher than WT) at intermediate NaCl concentrations (50–150 mM) (Figure 1B).

### *SAMdC1* Overexpression Alters Abscisic Acid Metabolism and Abscisic Acid-Responsive Gene Expression

**Figure 2:**
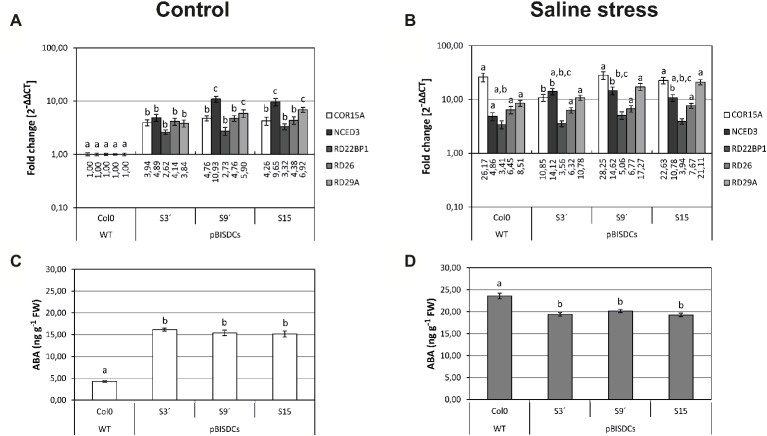
Effects of SAMDC1 overexpression on ABA metabolism and response. Four-week old Arabidopsis WT and pBISDCs transgenic plants overexpressing *SAMDC1* (S3′, S9, and S15), were watered with solution without (control) or with 250 mM NaCl (saline stress). Expression levels of a set of ABA biosynthesis and ABA-response related genes were determined in leaves by qRT-PCR after 6 h in control **(A)** or salt stress conditions **(B)**. For each gene, data are expressed as fold change relative to the level measured in WT plants in control conditions (2^−ΔΔCT^). ABA levels were also determined in the same leaves in control **(C)** and salt stress conditions **(D)**, expressed as ng (g FW)^−1^. Graph show the mean of three biological replicates ± standard deviation. Significant differences between plant lines are indicated with letters (ANOVA, Tukey HSD test, *p* < 0.05).

Given the previous result, a more specific search of genes related to the salt stress response within the transcriptome of the pBISDCs plants was carried out, locating a set of genes that code for proteins related to the ABA synthesis and response that showed significant expression changes compared to WT plants (Supplementary Figure S2). The expression of some of these ABA-related genes was checked by qRT-PCR to confirm the results observed in transcriptome studies. Expression levels of ABA-induced genes *COR15A* (AT2G42540; Baker et al., 1994), *RD26* (AT4G27410; Yamaguchi-Shinozaki et al., 1992), *RD29A* (AT5G52310; Yamaguchi-Shinozaki and Shinozaki, 1993), *RD22BP1* (AT1G32640; Abe et al., 2003) and *NCED3* (AT3G14440; Tan et al., 2003) were determined in 4-week old plants (Figure 2A). SAMDC1-overexpressing lines showed higher expression levels of all these ABA-related genes when compared to WT plants (Figure 2A). ABA-biosynthesis gene *NCED3* showed the most pronounced changes (5 to 10-fold).

Plants of same age were also exposed to salt stress. After 6 h of 250 mM NaCl exposure, the level of expression of all five ABA-related genes increased in WT plants (Figure 2B). Regarding pBISDCs transgenic lines response to NaCl exposure, *RD26* (all three lines) and *NCED3* (two lines) maintained expression levels similar to control conditions, while *COR15A* (two lines) raised to similar levels observed in WT plants exposed to stress. Also, *RD29A* (all lines) increased its expression in pBISDCs lines to higher levels than WT plants in salt stress conditions (Figure 2B).

ABA levels were also determined in both pBISDCs and WT lines in control and salt stress conditions. *SAMDC1-*overexpressing lines showed also higher ABA levels (about 3-fold) than WT lines on non-stressed 4-week old plants (Figure 2C). After 6 h of salt exposure, ABA levels raised in WT in greater extent than transgenic lines, reaching to a slightly higher level than in Spm-accumulating transgenic lines (Figure 2D). Similar results in gene expression and ABA levels were found for 5 day-old plate-grown seedlings and did not changed when photoperiod was set to short day (data not shown).

### Spm Treatment Raises ABA Levels and Induces ABA-Related Gene Expression

**Figure 3:**
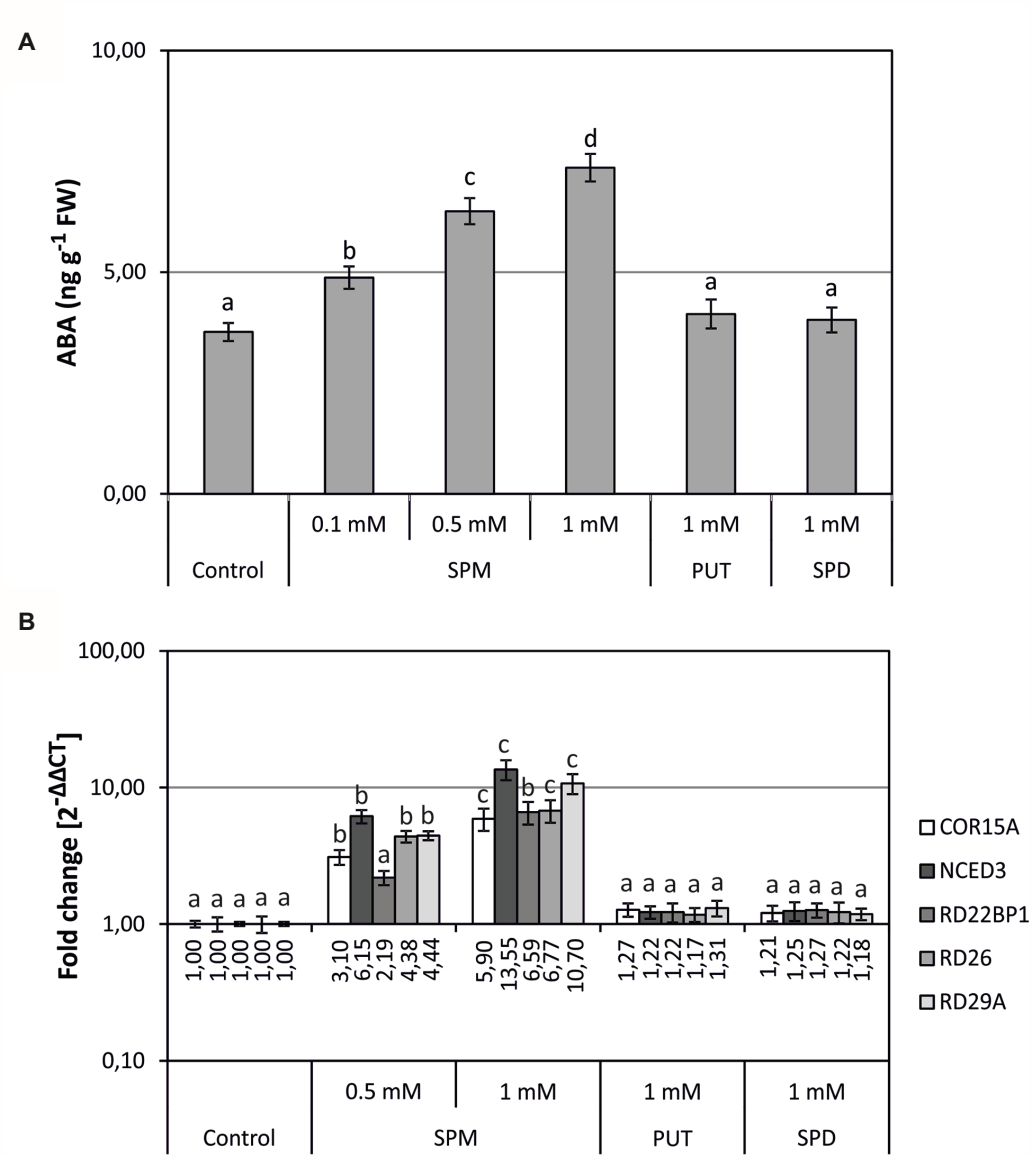
Effects of external PA treatment on ABA levels and ABA-related gene expression. Arabidopsis WT plants were grown for 5 days in plates supplemented with Put, Spd, or Spm, as well as in control plates without the amendment of PAs. **(A)** ABA levels, expressed as ng (g FW)^−1^. **(B)** Expression levels of ABA-responsive genes were determined by qRT-PCR. For each gene, data are expressed as fold change relative to the level measured in WT plants in control conditions (2^−ΔΔCT^). Graph show the mean of three biological replicates ± standard deviation. Significant differences between treatments are indicated with letters (ANOVA, Tukey HSD test, *p* < 0.05).

Results described above suggested a possible link between the observed differences in ABA and high Spm levels. To further test this possibility, PAs were exogenously supplied to plates where WT seeds were sown and let grow for 5 days. ABA levels increased in Spm-supplemented seedlings, with a positive correlation between ABA levels and external Spm concentration (Figure 3A). This positive correlation affected also to expression of the chosen set of ABA-related genes, with *NCED3* showing the most important induction in response to external Spm (Figure 3B). However, the growth of seedlings in the presence of external Put or Spd did not produce significant changes in ABA levels or in the expression of ABA-related genes (Figure 3).

### Spm Deficiency Affects Expression of Abscisic Acid-Induced Genes

**Figure 4:**
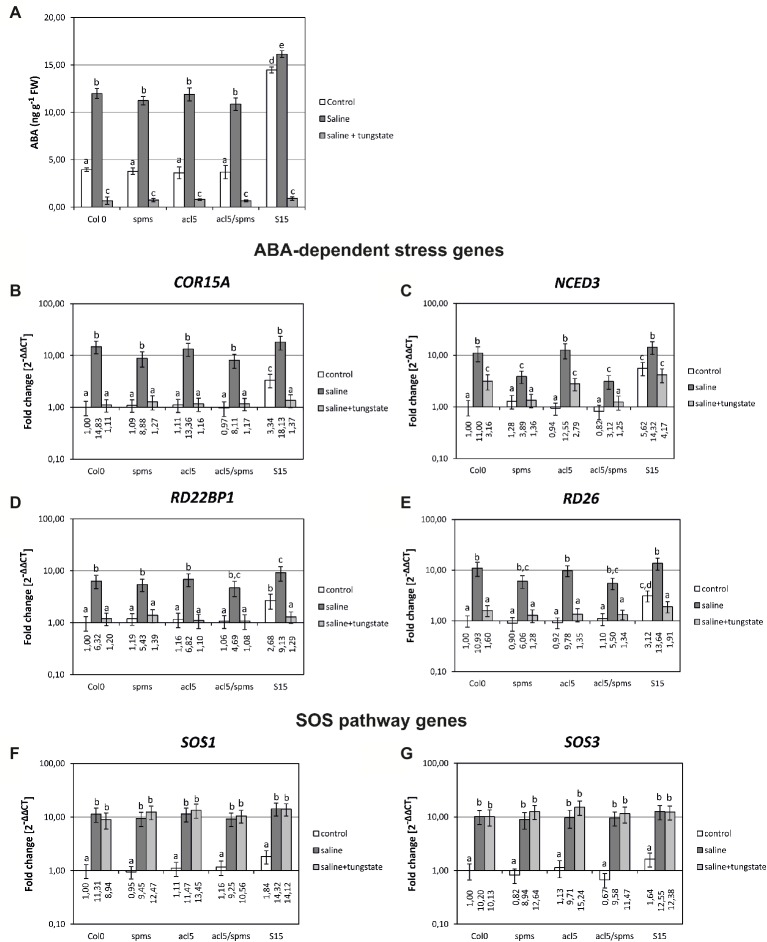
Effects of Spm levels and tungstate treatment on ABA levels and gene expression of salt stress response genes. Arabidopsis WT, *spms-1*, *acl5-1*, *acl5-1/spms1*, and pBISDCs-S15 plants were grown for 5 days in plates without (control), or supplemented with 250 mM NaCl (saline) or 250 mM NaCl plus 0.1 mM tungstate (saline + tungstate). ABA levels **(A)**, expressed as ng (g FW)^−1^, were measured for each plant and condition. Also, qRT-PCR was used to determine the expression levels of salt stress-related response genes in all conditions assayed **(B–G)**. For each gene, data are expressed as fold change relative to the level measured in WT plants in control conditions (2^−ΔΔCT^). Graph show the mean of three biological replicates ± standard deviation. Significant differences between treatments are indicated with letters (ANOVA, Tukey HSD test, *p* < 0.05).

Moreover, the possible relationship between ABA-related stress response and Spm levels was also tested in different Spm-deficiency scenarios. Therefore, ABA levels were determined in 5-day old plate-grown seedlings of tSpm synthase mutants (*acl5-1*), that have similar free Spm levels than WT plants (Hanzawa et al., 2000; Imai et al., 2004), as well as in *spms-1* mutants and double mutant *acl5-1/spms-1*, with no detectable levels of Spm (Figure 4A; Imai et al., 2004). In control conditions, all mutants showed similar ABA levels to those of WT seedlings. On the other hand, when seedlings were exposed to 250 mM NaCl for 6 h (salt stress conditions), ABA on *acl5-1* mutant rose to levels similar to those observed stressed WT seedlings. On the contrary, *spm-1* and *acl5-1/spm-1* mutants showed slightly lower ABA levels than WT stressed seedlings (Figure 4A). In the same conditions, Spm-accumulating line pBISDCs-S15 had the highest levels of ABA for both control and salt stress conditions (Figure 4A).

Expression of the ABA-responsive genes previously analyzed was also determined by qRT-PCR in Spm-deficient mutants (Figures 4B–E). Under control conditions, expression levels in the mutants were similar to those found in WT plants for all genes, but their expression pattern changed in salt exposed seedlings, depending on the mutant considered. Thus, *spms-1* and *acl5-1/spms-1* mutants were not able to raise *NCED3* and *RD26* expression when exposed to salt to the levels observed in salt-stressed WT and *acl5-1* seedlings (Figures 4C,E, respectively). Similar observations were made for *COR15A*, although the expression levels were more close to WT stressed seedlings (Figure 4B). On the other side, *RD22BP1* gene showed expression fold-change levels closer to the observed in WT salt-stressed seedlings for both *spms-1* mutants assayed (Figure 4D).

### Spm Is Able to Modulate Plant Responses to Salt Stress in an Abscisic Acid-Independent Way

**Figure 5:**
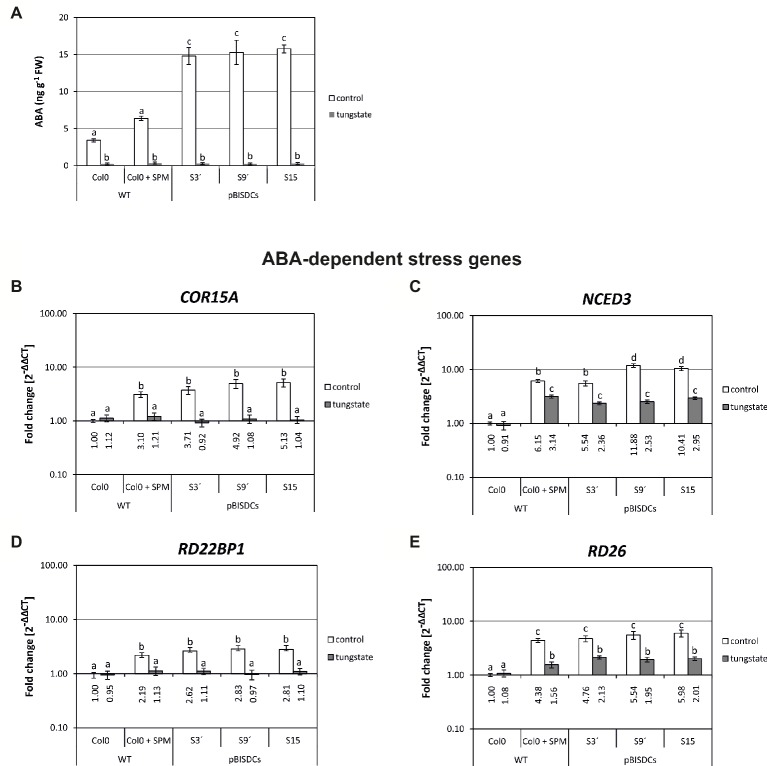
Effects of Spm levels, Spm treatment or tungstate treatment on ABA levels and gene expression of ABA-related genes. Arabidopsis WT, and pBISDCs transgenic plants overexpressing SAMDC1 (S3′, S9, and S15) plants were grown for 5 days in plates without (control), or supplemented with 0.1 mM tungstate (tungstate). Additionally, WT plants were grown in plates supplemented with 0.5 mM SPM. ABA levels **(A)**, expressed as ng (g FW)^−1^, were measured for each plant and condition. Also, qRT-PCR was used to determine the expression levels of ABA-related genes in all conditions assayed **(B–E)**. For each gene, data are expressed as fold change relative to the level measured in WT plants in control conditions (2^−ΔΔCT^). Graph show the mean of three biological replicates ± standard deviation. Significant differences between treatments are indicated with letters (ANOVA, Tukey HSD test, *p* < 0.05).

ABA synthesis was blocked by addition of 0.1 mM tungstate in plates. As expected, ABA levels dropped to levels <1 ng ABA g FW^−1^ in 5 days seedlings of WT, Spm-treated WT or pBISDCs plants (Figure 5A). Expression levels of *COR15A* and *RD22BP1* were higher in Spm-treated WT and pBISDCs lines than in WT seedlings (Figures 5B,D). In the presence of tungstate, expression of those genes was similar to the levels observed in the WT seedlings. (Fold changes ≤1) (Figures 5B,D), suggesting that their induction by Spm is determined by ABA-dependent pathways. On the contrary, *NCED3* expression levels in presence of tungstate remained higher in Spm-treated plants and pBISDCs lines than in WT plants (Figure 5C). *RD26* also showed maintenance of their expression after tungstate treatment combined with Spm or in pBISDCs seedlings (Figure 5E) but with fold change differences relative to WT control less pronounced than those observed for *NCDE3* (Figure 5E).

Once determined that higher Spm levels affect expression of ABA-related genes, we also tested the effect of blocking ABA biosynthesis in the different Spm-deficient situations described previously. Expression of the ABA dependent genes *COR15A*, *RD22BP1, NCED3*, and *RD26* was analyzed under control and salt stress conditio*ns* in the presence or absence of tungstate. Expression levels of *COR15A* and *RD22BP1* did not respond to salt stress in the presence of tungstate either in WT type, Spm-deficient mutants or the pBISDCs line S15 (Figures 4B,D). However, after salt stress, *NCDE3* rises to levels higher than control conditions in WT, the pBISDCs line S15 and *acl5-1* seedlings, and only dropped to similar levels observed in WT in Spm-deficient *spm-1* and *acl5-1/spm-1* mutants (Figure 4C). *RD26* also showed maintenance of their expression with tungstate treatment combined with salt stress, but with fold change differences relative to WT control less pronounced than those observed for *NCDE3* (Figure 4E). Similar results were obtained when ABA biosynthesis was blocked with Fluoridone (data not shown).

The specificity of Spm in relation with ABA-dependent stress responses was checked by analyzing the expression levels of *SOS1* (AT2G01980) and *SOS3* (AT5G24270), two members of the ABA-independent SOS (Salt Overly Sensitive) salt-signaling response pathway (Ji et al., 2013). *SOS1* and *SOS3*·expression was similar in all seedlings of the lines assayed, and salt stress induced their expression at similar levels independently of tungstate presence or the level of Spm in the seedlings (Figures 4F,G).

## Discussion

During the last years, the manipulation of polyamine levels by transgenic approaches or use of loss or gain mutants has proved a useful tool to gain knowledge about possible polyamine roles in plant processes (Alcazar et al., 2006b). Transgenic approaches include heterologous overexpression studies of *ODC*, *ADC*, *SAMDC*, and *SPDS* from different animal and plant sources in rice, tobacco, tomato, and Arabidopsis (Alcázar et al., 2010).

Likewise, Spm accumulation has been previously observed in transgenic rice plants constitutively overexpressing the *Datura stramonium* SAMDC cDNA in transgenic rice (Thu-Hang et al., 2002) or the yeast SAMDC in tobacco (Cheng et al., 2009), as well as using ABA inducible expression of Tritordeum SAMDC in rice (Roy and Wu, 2002) or ripening-induced expression of yeast SAMDC in tomato (Mehta et al., 2002), where accumulation of Spd and Spm was observed. High levels of Put, Spd, and Spm were also observed in tobacco plants that overexpress constitutively carnation SAMDC (Wi et al., 2006). In a previous work, we were able to describe that overexpression of *SAMDC1* gene in Arabidopsis leads to plants with higher Spm content than WT plants. Also, the transcriptome of pBISDCs transgenic lines showed an increase in the expression of a set of genes enriched in functional categories involved in defense-related processes against both biotic and abiotic stresses (Marco et al., 2011). In line with these results, Arabidopsis Spm-accumulating lines obtained by overexpression of SPMS share a common set of 234 genes that includes genes related with the response to water deprivation or cold acclimation (Gonzalez et al., 2011; Marco et al., 2011). In addition, it was previously observed that external Spm treatment modulated the expression of a large number of defense-related genes (Mitsuya et al., 2009). In fact, 28 genes induced from that study are also overexpressed in the pBISDCs transgenic lines, including the transcription factor AtbZIP60 (Iwata and Koizumi, 2005) and the mitogen-activated protein kinase AtMAPK3 (Takahashi et al., 2003). Moreover, Arabidopsis AtPO4-deficient plants show increased Spm levels in the roots and up-regulation of several genes encoding drought stress response proteins (Kamada-Nobusada et al., 2008).

The activation of this set of stress response genes due to Spm accumulation could be the factor that would explain the tolerance of those plants to salt stress (Figure 1). Absence of Spm on mutant line *acl5-1/spm-1* causes a defect of Ca^2+^ homeostasis and resulted in hypersensitivity to salt stress, being this phenotype mitigated only by exogenously applied Spm, but not by Spd or Put (Yamaguchi et al., 2006). On the other hand, increased tolerance to abiotic stress by increasing Spm levels had been previously observed in other transgenic plant systems. Rice plants over-expressing *Tritordeum* SAMDC under the control of an ABA inducible promoter accumulate Spd and Spm and are less sensitive to salt stress (Roy and Wu, 2002). Also, rice plants with constitutive expression of *D. stramonium SAMDC* showed increased levels of Spm and an improved recovery after exposure to drought conditions (Peremarti et al., 2009). In the same trend, over-expression of carnation SAMDC produced accumulation of total PAs in tobacco, and generated a broad-spectrum tolerance to abiotic stresses (Wi et al., 2006). More recently, is has been described that constitutive overexpression of *Capsicum annuum S*AMDC in Arabidopsis increases Spd and Spm levels and leads to an increased drought tolerance of the transgenic plants compared to WT (Wi et al., 2014). Raise of Spm and Spd levels was also obtained in Arabidopsis plants by overexpression of cucurbita *SPDS* gene, leading to an enhanced tolerance to multiple environmental stresses (Kasukabe et al., 2004). Conversely, alterations of Spm levels have also been reported by downregulation of SAMDC gene by RNA interference strategies in tobacco (Moschou et al., 2008) and rice (Chen et al., 2014), leading to plants with reduced Spd and Spm levels and an enhanced salinity-induced programmed cell death in tobacco (Moschou et al., 2008) or with a reduced tolerance to stress by drought, salinity or chilling in rice (Chen et al., 2014).

The relationship among PA metabolism, abiotic stress, and ABA has been previously reported in several studies. Expression of *ADC2*, *SPMS*, and *SAMDC2* genes is induced by the exogenous application of ABA in Arabidopsis (Urano et al., 2003). Also, induction of *ADC2*, *SPDS1*, and *SPMS* by drought stress is Arabidopsis is an ABA-dependent response, since up-regulation is not observed in ABA deficient (*aba2*) and insensitive (*abi1*) mutants (Alcazar et al., 2006a). Moreover, maize *ADC2*, *ZmSPDS1* and *ZmSPDS2* genes are also induced by NaCl and ABA treatments (Jiménez-Bremont et al., 2007).

Based on these results, the effect of Spm accumulation on the expression of ABA-related genes in pBISDCs plants was examined more in depth with the MAPMAN tool (Supplementary Figure S2; Usadel et al., 2005). We found three upregulated genes (*NCED3*, *NCED4*, and *ABA2*) that code for ABA biosynthesis enzymes (González-Guzmán et al., 2002; Tan et al., 2003). In addition, other genes coding for ABA-induced proteins like ATHVA22A, ATHVA22B (Chen et al., 2002), KIN1 (Wang et al., 1995) or the gram-domain containing protein GER5 (AT5G13200; Baron et al., 2014) were also up-regulated (Supplementary Figure S2, overexpressed). The set of under-expressed genes also includes some ABA-responsive genes as AAO2 (Seo et al., 2000), ABF4 (Kang et al., 2002) and a couple more of ABA-responsive proteins. (Supplementary Figure S2, under-expressed). These observations pointed us to carry out a more detailed study of the ABA levels and ABA response in pBISDCs plants. In order to confirm the expression data observed in the transcriptome study, expression levels of several ABA-related genes were checked by qRT-PCR. pBISDCs lines have higher levels of expression of genes that code for ABA biosynthesis genes (Supplementary Figure S2), including *NCED3* (Figure 2A), and consequently higher ABA levels than WT lines in control conditions (Figure 2C).

Expression levels of a set of ABA-responsive genes were also checked by qRT-PCR. Cold-Regulated 15A gene (*COR15A*) codes for a member of the Late Embryogenesis Abundant protein family with a role in the protection of the chloroplast structures during freeze-induced dehydration (Steponkus et al., 1998). Responsive to Desiccation 26 gene (*RD26*) is induced in response to desiccation, and encodes a transcriptional activator that acts in ABA-mediated dehydration response (Yamaguchi-Shinozaki et al., 1992; Song et al., 2016). *RD22BP1* gene also encodes a MYC-related transcriptional activator that is induced by dehydration stress and ABA treatment (Abe et al., 2003; Liu et al., 2018). *RD29A* encodes a hydrophilic protein of unknown function that is induced by salt and drought stresses (Yamaguchi-Shinozaki and Shinozaki, 1993). Expression levels of all these ABA-dependent genes were increased in the pBISDCs lines (Figure 2A). Induction of *RD26* and *COR15A* has been previously observed in Arabidopsis plants overexpressing cucurbita *SPDS*, with increased levels of Spd and Spm (Kasukabe et al., 2004). Higher ABA levels on pBISDCs lines could also explain the activation of at least part of the stress-related genes observed in these Spm-accumulating lines (Supplementary Figure S2).

Additionally, external application of Spm to WT plants lead to similar changes in *NCDE3* expression, ABA levels, and expression levels of the other ABA-responsive genes (Figure 5). Moreover, Spm-deficient mutants (*spm-1*, *acl5-1/spm-1*) were not able to raise ABA to similar levels than WT plants when exposed to salt stress (Figure 4A). Same observation was done for the expression levels of genes *NCED3* (Figure 4C) and *RD26* (Figure 4E). An increase in ABA levels has been also reported in soybean seeds treated with Spm (Radhakrishnan and Lee, 2013).

Finally, when ABA synthesis was blocked with tungstate, *NCED3* maintained a certain level of induction in plants with normal (WT, *acl5-1*) or high (Spm-treated WT and pBISDCs lines) levels of Spm, whereas this was not observed in Spm-deficient mutants *spm-1* and *acl5-1/spm-1* (Figure 5, tungstate and Figure 4, saline + tungstate). The existence of an ABA-independent pathway responsible for part of the induction of ABA biosynthesis genes by salt stress was proposed previously (Barrero et al., 2006). Those authors pointed out that severe ABA-deficient mutants still showed a NaCl-dependent induction of *NCED3*, *AAO3*, and *ABA1*, being *NCED3* the gene that showed a stronger induction with NaCl in ABA absence. Taken together, our results suggest that Spm could have a possible role in the induction of the expression of *NCED3* by this ABA-independent pathway salt stress response.

On the other hand, expression levels of two members of the SOS signaling pathway, involved in the maintenance of ion homeostasis during salt stress (Ji et al., 2013) are not altered in Spm-accumulating lines in response to NaCl stress (Figures 4F,G). This observation was previously reported in Spm-deficient mutants (Yamaguchi et al., 2006).

In summary, the results obtained in this study add more evidences to the involvement of Spm in plant stress responses, for which various protective roles have been proposed. Our results suggest that one of these mechanisms could involve the modulation of ABA levels in salt stress response through modulation of ABA biosynthesis by affecting *NCED3* gene expression. At the same time, Spm could be involved in an ABA-independent stress response pathway, as suggested by the results observed when ABA biosynthesis is blocked with tungstate. It remains to be determined which other changes in gene expression observed in Spm-accumulating plants results from the direct action of Spm or which gene expression changes are the consequence of cross-talking between Spm and other stress-response signaling pathways, including ABA.

## Data Availability

All datasets generated for this study are included in the manuscript and/or the Supplementary Files.

## Author Contributions

FM, EB and PC conceived the experimental design. FM and EB conducted the experiments. EB and TL conducted ABA measurements FM, EB and PC conducted gene selection and primer design. FM and PC wrote the manuscript.

### Conflict of Interest Statement

The authors declare that the research was conducted in the absence of any commercial or financial relationships that could be construed as a potential conflict of interest.

## References

[ref1] Abe H. Urao T. Ito T. Seki M. Shinozaki K. Yamaguchi-Shinozaki K. (2003). Arabidopsis AtMYC2 (bHLH) and AtMYB2 (MYB) function as transcriptional activators in abscisic acid signaling. Plant Cell 15, 63–78. 10.1105/tpc.006130 12509522 PMC143451

[ref2] Alcázar R. Altabella T. Marco F. Bortolotti C. Reymond M. Koncz C. (2010). Polyamines: molecules with regulatory functions in plant abiotic stress tolerance. Planta 231, 1237–1249. 10.1007/s00425-010-1130-0 20221631

[ref3] Alcazar R. Cuevas J. C. Patron M. Altabella T. Tiburcio A. F. (2006a). Abscisic acid modulates polyamine metabolism under water stress in *Arabidopsis thaliana*. Physiol. Plant. 128, 448–455. 10.1111/j.1399-3054.2006.00780.x

[ref4] Alcazar R. Marco F. Cuevas J. C. Patron M. Ferrando A. Carrasco P. (2006b). Involvement of polyamines in plant response to abiotic stress. Biotechnol. Lett. 28, 1867–1876. 10.1007/s10529-006-9179-3 17028780

[ref5] An Y. Q. McDowell J. M. Huang S. McKinney E. C. Chambliss S. Meagher R. B. (1996). Strong, constitutive expression of the Arabidopsis ACT2/ACT8 actin subclass in vegetative tissues. Plant J. 10, 107–121. 10.1046/j.1365-313X.1996.10010107.x 8758981

[ref6] Baker S. S. Wilhelm K. S. Thomashow M. F. (1994). The 5′-region of *Arabidopsis thaliana* cor15a has cis-acting elements that confer cold-, drought- and ABA-regulated gene expression. Plant Mol. Biol. 24, 701–713. 10.1007/BF00029852, PMID: 8193295

[ref7] Baron K. N. Schroeder D. F. Stasolla C. (2014). GEm-Related 5 (GER5), an ABA and stress-responsive GRAM domain protein regulating seed development and inflorescence architecture. Plant Sci. 223, 153–166. 10.1016/j.plantsci.2014.03.017 24767125

[ref8] Barrero J. M. Rodriguez P. L. Quesada V. Piqueras P. Ponce M. R. Micol J. L. (2006). Both abscisic acid (ABA)-dependent and ABA-independent pathways govern the induction of NCED3, AAO3 and ABA1 in response to salt stress. Plant Cell Environ. 29, 2000–2008. 10.1111/j.1365-3040.2006.01576.x, PMID: 16930325

[ref9] Bouchereau A. Aziz A. Larher F. Martin-Tanguy J. (1999). Polyamines and environmental challenges: recent development. Plant Sci. 140, 103–125. 10.1016/S0168-9452(98)00218-0

[ref10] Chen M. Chen J. Fang J. Guo Z. Lu S. (2014). Down-regulation of S-adenosylmethionine decarboxylase genes results in reduced plant length, pollen viability, and abiotic stress tolerance. Plant Cell, Tissue Organ Cult. 116, 311–322. 10.1007/s11240-013-0405-0

[ref11] Chen C. N. Chu C. C. Zentella R. Pan S. M. Ho T. H. (2002). AtHVA22 gene family in Arabidopsis: phylogenetic relationship, ABA and stress regulation, and tissue-specific expression. Plant Mol. Biol. 49, 633–644. 10.1023/A:1015593715144 12081371

[ref12] Cheng L. Zou Y. Ding S. Zhang J. Yu X. Cao J. (2009). Polyamine accumulation in transgenic tomato enhances the tolerance to high temperature stress. J. Integr. Plant Biol. 51, 489–499. 10.1111/j.1744-7909.2009.00816.x 19508360

[ref13] Cona A. Rea G. Angelini R. Federico R. Tavladoraki P. (2006). Functions of amine oxidases in plant development and defence. Trends Plant Sci. 11, 80–88. 10.1016/j.tplants.2005.12.009 16406305

[ref14] Cuevas J. C. Lopez-Cobollo R. Alcazar R. Zarza X. Koncz C. Altabella T.. (2008). Putrescine is involved in Arabidopsis freezing tolerance and cold acclimation by regulating abscisic acid levels in response to low temperature. Plant Physiol. 148, 1094–1105. 10.1104/pp.108.122945, PMID: 18701673 PMC2556839

[ref15] Fujita Y. Fujita M. Shinozaki K. Yamaguchi-Shinozaki K. (2011). ABA-mediated transcriptional regulation in response to osmotic stress in plants. J. Plant Res. 124, 509–525. 10.1007/s10265-011-0412-3 21416314

[ref16] Gill S. Tuteja N. (2010). Polyamines and abiotic stress tolerance in plants. Plant Signal. Behav. 5, 26–33. 10.4161/psb.5.1.10291 20592804 PMC2835953

[ref17] Gonzalez M. E. Marco F. Minguet E. G. Carrasco Sorli P. Blázquez M. A. Carbonell J. (2011). Perturbation of spermine synthase gene expression and transcript profiling provide new insights on the role of the tetraamine spermine in *Arabidopsis thaliana* defense against *Pseudomonas viridiflava*. Plant Physiol. 156, 2266–2277. 10.1104/pp.110.171413 21628628 PMC3149955

[ref18] González-Guzmán M. Apostolova N. Bellés J. M. Barrero J. M. Piqueras P. Ponce M. R. (2002). The short-chain alcohol dehydrogenase ABA2 catalyzes the conversion of xanthoxin to abscisic aldehyde. Plant Cell 14, 1833–1846. 10.1105/tpc.002477 12172025 PMC151468

[ref19] Hanzawa Y. Imai A. Michael A. J. Komeda Y. Takahashi T. (2002). Characterization of the spermidine synthase-related gene family in *Arabidopsis thaliana*. FEBS Lett. 527, 176–180. 10.1016/S0014-5793(02)03217-9, PMID: 12220656

[ref20] Hanzawa Y. Takahashi T. Komeda Y. (1997). ACL5: an Arabidopsis gene required for internodal elongation after flowering. Plant J. 12, 863–874. 10.1046/j.1365-313X.1997.12040863.x, PMID: 9375398

[ref21] Hanzawa Y. Takahashi T. Michael A. J. Burtin D. Long D. Pineiro M. (2000). ACAULIS5, an Arabidopsis gene required for stem elongation, encodes a spermine synthase. EMBO J. 19, 4248–4256. 10.1093/emboj/19.16.4248 10944107 PMC302034

[ref22] Imai A. Akiyama T. Kato T. Sato S. Tabata S. Yamamoto K. T. (2004). Spermine is not essential for survival of Arabidopsis. FEBS Lett. 556, 148–152. 10.1016/s0014-5793(03)01395-4 14706842

[ref23] Iuchi S. Kobayashi M. Taji T. Naramoto M. Seki M. Kato T.. (2001). Regulation of drought tolerance by gene manipulation of 9-cis-epoxycarotenoid dioxygenase, a key enzyme in abscisic acid biosynthesis in Arabidopsis. Plant J. 27, 325–333. 10.1046/j.1365-313x.2001.01096.x, PMID: 11532178

[ref24] Iwata Y. Koizumi N. (2005). An Arabidopsis transcription factor, AtbZIP60, regulates the endoplasmic reticulum stress response in a manner unique to plants. Proc. Natl. Acad. Sci. USA 102, 5280–5285. 10.1073/pnas.0408941102, PMID: 15781873 PMC555978

[ref25] Ji H. Pardo J. M. Batelli G. Van Oosten M. J. Bressan R. A. Li X. (2013). The salt overly sensitive (SOS) pathway: established and emerging roles. Mol. Plant 6, 275–286. 10.1093/mp/sst017 23355543

[ref26] Jiménez-Bremont J. F. Ruiz O. A. Rodríguez-Kessler M. (2007). Modulation of spermidine and spermine levels in maize seedlings subjected to long-term salt stress. Plant Physiol. Biochem. 45, 812–821. 10.1016/j.plaphy.2007.08.001 17890098

[ref520] Kamada-Nobusada T. Hayashi M. Fukazawa M. Sakakibara H. Nishimura M. (2008). A Putative Peroxisomal Polyamine Oxidase, AtPAO4, is Involved in Polyamine Catabolism in *Arabidopsis thaliana*. Plant Cell Physiol. 49, 1272–1282. 10.1093/pcp/pcn114, PMID: 18703589

[ref27] Kang J.-Y. Choi H.-I. Im M.-Y. Kim S. Y. (2002). Arabidopsis basic leucine zipper proteins that mediate stress-responsive abscisic acid signaling. Plant Cell 14, 343–357. 10.1105/tpc.010362, PMID: 11884679 PMC152917

[ref28] Kasukabe Y. He L. Nada K. Misawa S. Ihara I. Tachibana S. (2004). Overexpression of spermidine synthase enhances tolerance to multiple environmental stresses and up-regulates the expression of various stress-regulated genes in transgenic *Arabidopsis thaliana*. Plant Cell Physiol. 45, 712–722. 10.1093/pcp/pch083, PMID: 15215506

[ref29] Kende H. Zeevaart J. (1997). The five “classical” plant hormones. Plant Cell 9, 1197–1210. 10.1105/tpc.9.7.1197, PMID: 12237383 PMC156991

[ref30] Knott J. M. Römer P. Sumper M. (2007). Putative spermine synthases from *Thalassiosira pseudonana* and *Arabidopsis thaliana* synthesize thermospermine rather than spermine. FEBS Lett. 581, 3081–3086. 10.1016/j.febslet.2007.05.074, PMID: 17560575

[ref31] Kusano T. Berberich T. Tateda C. Takahashi Y. (2008). Polyamines: essential factors for growth and survival. Planta 228, 367–381. 10.1007/s00425-008-0772-7 18594857

[ref32] Liu S. Lv Z. Liu Y. Li L. Zhang L. (2018). Network analysis of ABA-dependent and ABA-independent drought responsive genes in *Arabidopsis thaliana*. Genet. Mol. Biol. 41, 624–637. 10.1590/1678-4685-gmb-2017-0229, PMID: 30044467 PMC6136374

[ref33] Livak K. J. Schmittgen T. D. (2001). Analysis of relative gene expression data using real-time quantitative PCR and the 2-[Delta][Delta]CT Method. Methods 25, 402–408. 10.1006/meth.2001.1262, PMID: 11846609

[ref34] Marco F. Alcazar R. Tiburcio A. F. Carrasco P. (2011). Interactions between polyamines and abiotic stress pathway responses unraveled by transcriptome analysis of polyamine overproducers. OMICS 15, 775–781. 10.1089/omi.2011.0084 22011340 PMC3229227

[ref35] Marin E. Nussaume L. Quesada A. Gonneau M. Sotta B. Hugueney P. (1996). Molecular identification of zeaxanthin epoxidase of *Nicotiana plumbaginifolia*, a gene involved in abscisic acid biosynthesis and corresponding to the ABA locus of *Arabidopsis thaliana*. EMBO J. 15, 2331–2342. 8665840 PMC450162

[ref36] Mehta R. A. Cassol T. Li N. Ali N. Handa A. K. Mattoo A. K. (2002). Engineered polyamine accumulation in tomato enhances phytonutrient content, juice quality, and vine life. Nat. Biotechnol. 20, 613–618. 10.1038/nbt0602-613 12042867

[ref37] Minocha R. Majumdar R. Minocha S. C. (2014). Polyamines and abiotic stress in plants: a complex relationship. Front. Plant Sci. 5:175. 10.3389/fpls.2014.00175 24847338 PMC4017135

[ref38] Mitsuya Y. Takahashi Y. Berberich T. Miyazaki A. Matsumura H. Takahashi H.. (2009). Spermine signaling plays a significant role in the defense response of *Arabidopsis thaliana* to cucumber mosaic virus. J. Plant Physiol. 166, 626–643. 10.1016/j.jplph.2008.08.006, PMID: 18922600

[ref39] Moschou P. N. Paschalidis K. A. Delis I. D. Andriopoulou A. H. Lagiotis G. D. Yakoumakis D. I.. (2008). Spermidine exodus and oxidation in the apoplast induced by abiotic stress is responsible for H_2_O_2_ signatures that direct tolerance responses in tobacco. Plant Cell 20, 1708–1724. 10.1105/tpc.108.059733, PMID: 18577660 PMC2483379

[ref40] Moschou P. N. Wu J. Cona A. Tavladoraki P. Angelini R. Roubelakis-Angelakis K. A. (2012). The polyamines and their catabolic products are significant players in the turnover of nitrogenous molecules in plants. J. Exp. Bot. 63, 5003–5015. 10.1093/jxb/ers202, PMID: 22936828

[ref41] Murashige T. Skoog F. (1962). A revised medium for rapid growth and bio assays with tobacco tissue cultures. Physiol. Plant. 15, 473–497. 10.1111/j.1399-3054.1962.tb08052.x

[ref42] Norman S. M. Poling S. M. Maier V. P. (1988). An indirect enzyme-linked immunosorbent assay for (+)-abscisic acid in Citrus, Ricinus, and Xanthium leaves. J. Agric. Food Chem. 36, 225–231. 10.1021/jf00079a056

[ref43] Peremarti A. Bassie L. Christou P. Capell T. (2009). Spermine facilitates recovery from drought but does not confer drought tolerance in transgenic rice plants expressing *Datura stramonium* S-adenosylmethionine decarboxylase. Plant Mol. Biol. 70, 253–264. 10.1007/s11103-009-9470-5 19234674

[ref44] Planas-Portell J. Gallart M. Tiburcio A. F. Altabella T. (2013). Copper-containing amine oxidases contribute to terminal polyamine oxidation in peroxisomes and apoplast of *Arabidopsis thaliana*. BMC Plant Biol. 13:109. 10.1186/1471-2229-13-109, PMID: 23915037 PMC3751259

[ref45] Radhakrishnan R. Lee I.-J. (2013). Ameliorative effects of spermine against osmotic stress through antioxidants and abscisic acid changes in soybean pods and seeds. Acta Physiol. Plant. 35, 263–269. 10.1007/s11738-012-1072-1

[ref46] Roy M. Wu R. (2002). Overexpression of S-adenosylmethionine decarboxylase gene in rice increases polyamine level and enhances sodium chloride-stress tolerance. Plant Sci. 163, 987–992. 10.1016/S0168-9452(02)00272-8

[ref47] Sah S. K. Reddy K. R. Li J. (2016). Abscisic acid and abiotic stress tolerance in crop plants. Front. Plant Sci. 7, 571–571. 10.3389/fpls.2016.00571, PMID: 27200044 PMC4855980

[ref48] Schwartz S. H. Tan B. C. Gage D. A. Zeevaart J. A. D. McCarty D. R. (1997). Specific oxidative cleavage of carotenoids by VP14 of maize. Science 276, 1872–1874. 10.1126/science.276.5320.1872, PMID: 9188535

[ref49] Seki M. Narusaka M. Ishida J. Nanjo T. Fujita M. Oono Y. (2002). Monitoring the expression profiles of 7000 Arabidopsis genes under drought, cold and high-salinity stresses using a full-length cDNA microarray. Plant J. 31, 279–292. 10.1046/j.1365-313X.2002.01359.x 12164808

[ref50] Seo M. Koiwai H. Akaba S. Komano T. Oritani T. Kamiya Y. (2000). Abscisic aldehyde oxidase in leaves of *Arabidopsis thaliana*. Plant J. 23, 481–488. 10.1046/j.1365-313x.2000.00812.x 10972874

[ref51] Song L. Huang S. C. Wise A. Castanon R. Nery J. R. Chen H.. (2016). A transcription factor hierarchy defines an environmental stress response network. Science 354:aag1550. 10.1126/science.aag1550, PMID: 27811239 PMC5217750

[ref52] Steponkus P. L. Uemura M. Joseph R. A. Gilmour S. J. Thomashow M. F. (1998). Mode of action of the COR15a gene on the freezing tolerance of *Arabidopsis thaliana*. Proc. Natl. Acad. Sci. USA 95, 14570–14575. 10.1073/pnas.95.24.14570 9826741 PMC24414

[ref53] Takahashi Y. Berberich T. Miyazaki A. Seo S. Ohashi Y. Kusano T. (2003). Spermine signalling in tobacco: activation of mitogen-activated protein kinases by spermine is mediated through mitochondrial dysfunction. Plant J. 36, 820–829. 10.1046/j.1365-313X.2003.01923.x, PMID: 14675447

[ref54] Takahashi Y. Cong R. Sagor G. H. Niitsu M. Berberich T. Kusano T. (2010). Characterization of five polyamine oxidase isoforms in *Arabidopsis thaliana*. Plant Cell Rep. 29, 955–965. 10.1007/s00299-010-0881-1, PMID: 20532512

[ref55] Tan B.-C. Joseph L. M. Deng W.-T. Liu L. Li Q.-B. Cline K.. (2003). Molecular characterization of the Arabidopsis 9-cis epoxycarotenoid dioxygenase gene family. Plant J. 35, 44–56. 10.1046/j.1365-313X.2003.01786.x, PMID: 12834401

[ref56] Thompson A. J. Jackson A. C. Symonds R. C. Mulholland B. J. Dadswell A. R. Blake P. S. (2000). Ectopic expression of a tomato 9-cis-epoxycarotenoid dioxygenase gene causes over-production of abscisic acid. Plant J. 23, 363–374. 10.1046/j.1365-313x.2000.00789.x 10929129

[ref57] Thu-Hang P. Bassie L. Safwat G. Trung-Nghia P. Christou P. Capell T. (2002). Expression of a heterologous S-adenosylmethionine decarboxylase cDNA in plants demonstrates that changes in S-adenosyl-L-methionine decarboxylase activity determine levels of the higher polyamines spermidine and spermine. Plant Physiol. 129, 1744–1754. 10.1104/pp.010966, PMID: 12177487 PMC166762

[ref58] Tiburcio A. F. Altabella T. Bitrián M. Alcázar R. (2014). The roles of polyamines during the lifespan of plants: from development to stress. Planta 240, 1–18. 10.1007/s00425-014-2055-9 24659098

[ref59] Tiburcio A. F. Kaur-Sawhney R. Galston A. W. (1990). “Polyamine metabolism” in The biochemistry of plants, intermediary nitrogen metabolism. eds. Mifflin B. J. Lea P. J. (New York: Academic Press), 283–325.

[ref60] Tuteja N. Sopory S. K. (2008). Chemical signaling under abiotic stress environment in plants. Plant Signal. Behav. 3, 525–536. 10.4161/psb.3.8.6186 19513246 PMC2634487

[ref61] Urano K. Hobo T. Shinozaki K. (2005). Arabidopsis ADC genes involved in polyamine biosynthesis are essential for seed development. FEBS Lett. 579, 1557–1564. 10.1016/j.febslet.2005.01.048 15733873

[ref62] Urano K. Yoshiba Y. Nanjo T. Igarashi Y. Seki M. Sekiguchi F. (2003). Characterization of Arabidopsis genes involved in biosynthesis of polyamines in abiotic stress responses and developmental stages. Plant Cell Environ. 26, 1917–1926. 10.1046/j.1365-3040.2003.01108.x

[ref63] Usadel B. Nagel A. Thimm O. Redestig H. Blaesing O. Palacios-Rojas N.. (2005). Extension of the visualization tool MapMan to allow statistical analysis of arrays, display of coresponding genes, and comparison with known responses. Plant Physiol. 138, 1195–1204. 10.1104/pp.105.060459, PMID: 16009995 PMC1176394

[ref64] Vishwakarma K. Upadhyay N. Kumar N. Yadav G. Singh J. Mishra R. K. (2017). Abscisic acid signaling and abiotic stress tolerance in plants: a review on current knowledge and future prospects. Front. Plant Sci. 8:161. 10.3389/fpls.2017.00161 28265276 PMC5316533

[ref65] Walker-Simmons M. (1987). ABA levels and sensitivity in developing wheat embryos of sprouting resistant and susceptible cultivars. Plant Physiol. 84, 61–66. 10.1104/pp.84.1.61 16665406 PMC1056528

[ref66] Wang H. Datla R. Georges F. Loewen M. Cutler A. J. (1995). Promoters from kin1 and cor6.6, two homologous *Arabidopsis thaliana* genes: transcriptional regulation and gene expression induced by low temperature, ABA, osmoticum and dehydration. Plant Mol. Biol. 28, 605–617. 7647294 10.1007/BF00021187

[ref530] Weiler E. W. (1980). Radioimmunoassays for the differential and direct analysis of free and conjugated abscisic acid in plant extracts. Planta 148, 262–272. 10.1007/BF00380037 24309829

[ref67] Wi S. J. Kim S. J. Kim W. T. Park K. Y. (2014). Constitutive S-adenosylmethionine decarboxylase gene expression increases drought tolerance through inhibition of reactive oxygen species accumulation in Arabidopsis. Planta 239, 979–988. 10.1007/s00425-014-2027-0 24477528

[ref68] Wi S. Kim W. Park K. (2006). Overexpression of carnation S-adenosylmethionine decarboxylase gene generates a broad-spectrum tolerance to abiotic stresses in transgenic tobacco plants. Plant Cell Rep. 25, 1111–1121. 10.1007/s00299-006-0160-3, PMID: 16642382

[ref69] Yamaguchi K. Takahashi Y. Berberich T. Imai A. Miyazaki A. Takahashi T. (2006). The polyamine spermine protects against high salt stress in *Arabidopsis thaliana*. FEBS Lett. 580, 6783–6788. 10.1016/j.febslet.2006.10.078 17140566

[ref70] Yamaguchi K. Takahashi Y. Berberich T. Imai A. Takahashi T. Michael A. J.. (2007). A protective role for the polyamine spermine against drought stress in Arabidopsis. Biochem. Biophys. Res. Commun. 352, 486–490. 10.1016/j.bbrc.2006.11.041, PMID: 17118338

[ref71] Yamaguchi-Shinozaki K. Koizumi M. Urao S. Shinozaki K. (1992). Molecular cloning and characterization of 9 cDNAs for genes that are responsive to desiccation in *Arabidopsis thaliana*: sequenceanalysis of one cDNA clone that encodes a putative transmembrane channel protein. Plant Cell Physiol. 33, 217–224. 10.1093/oxfordjournals.pcp.a078243

[ref72] Yamaguchi-Shinozaki K. Shinozaki K. (1993). Characterization of the expression of a desiccation-responsive rd29 gene of *Arabidopsis thaliana* and analysis of its promoter in transgenic plants. Mol. Gen. Genet. MGG 236, 331–340. 10.1007/BF00277130, PMID: 8437577

[ref73] Zarza X. Atanasov K. E. Marco F. Arbona V. Carrasco P. Kopka J. (2017). Polyamine oxidase 5 loss-of-function mutations in *Arabidopsis thaliana* trigger metabolic and transcriptional reprogramming and promote salt stress tolerance. Plant Cell Environ. 40, 527–542. 10.1111/pce.12714 26791972

