# Supplementary material for: Spermine Confers Stress Resilience by Modulating Abscisic Acid Biosynthesis and Stress Responses in Arabidopsis Plants
Source: Front Plant Sci. 2019 Jul 31;10:972. doi: 10.3389/fpls.2019.00972 (PMC6684778; doi:10.3389/fpls.2019.00972)
Supplement: Supplementary file 1 [file Data_Sheet_1.PDF]

SUPPLEMENTARY INFORMATION

Spermine confers stress resilience by modulating ABA biosynthesis and stress responses in Arabidopsis plants

Francisco Marco[1⁋]*, Enrique Busó[2,3⁋], Teresa Lafuente[4], Pedro Carrasco[2*]

[1]Departament de Biologia Vegetal, Universitat de València. E-46100, Burjassot, València, Spain.

[2]Departament de Bioquímica i Biologia Molecular. Universitat de València. E-46100, Burjassot, València, Spain.

[3]Current Adress:UCIM, Universitat de València. E-46021, València, Spain.

[4]Instituto de Agroquímica y Tecnología de Alimentos, CSIC, Paterna, València, Spain.

⁋ Both authors contributed equally to this work

*** Correspondence:** Pedro.Carrasco@uv.es

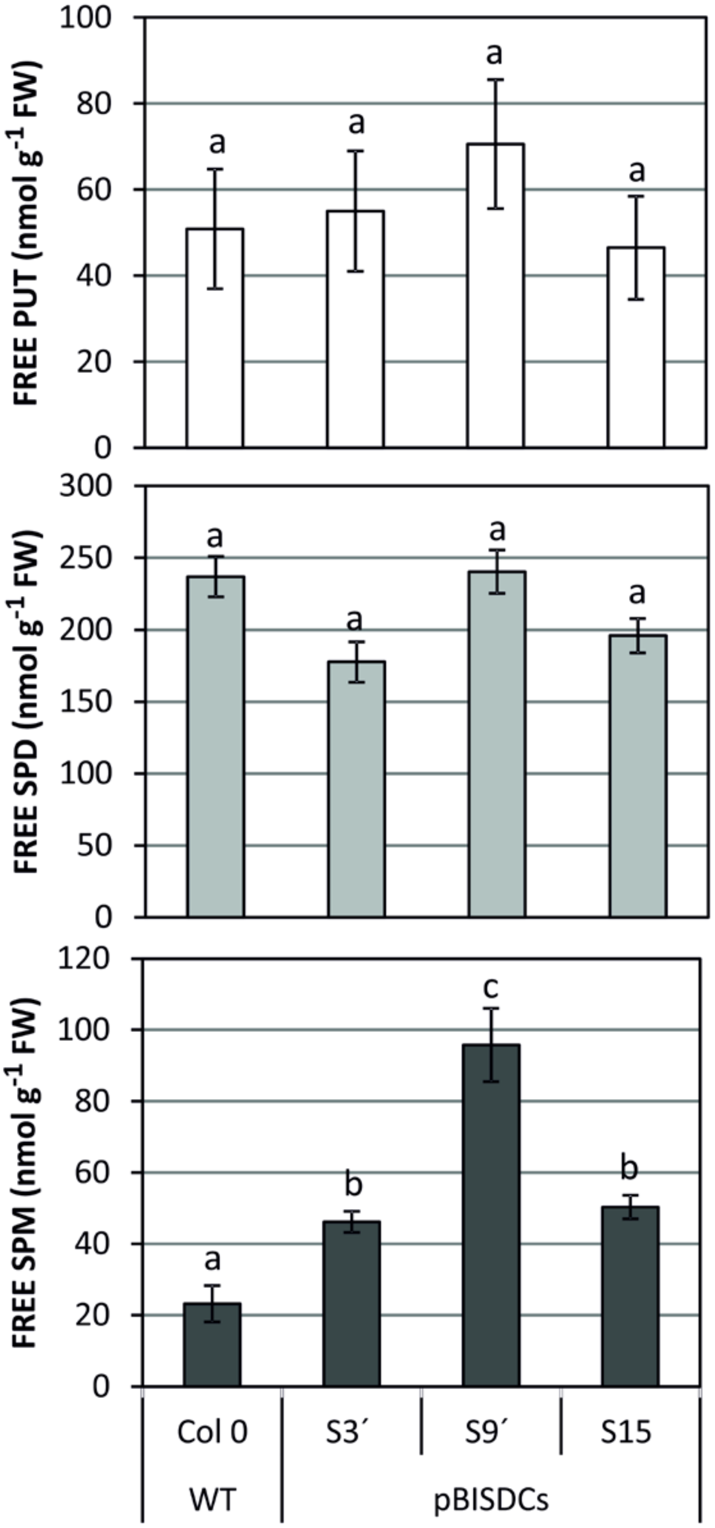

Figure S1. Free polyamine levels in 4-week-old Arabidopsis WT and pBISDCs transgenic plants overexpressing SAMDC1 (S3', S9 and S15), expressed as nmol (g FW)$^{-1}$. Graph show the mean of three biological replicates ± standard deviation. Significant differences between plant lines are indicated with letters (ANOVA, Tukey HSD test, $p < 0.05$). FW, fresh weight.

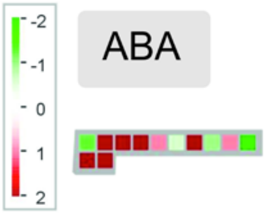

Overexpressed

AGI	Description	Log2 Fold Change
AT3G21780	UGT71B6 (UDP-GLUCOSYL TRANSFERASE 71B6)	6
AT4G19170	NCED4 (NINE-CIS-EPOXYCAROTENOID DIOXYGENASE 4)	4
AT3G14440	NCED3 (NINE-CIS-EPOXYCAROTENOID DIOXYGENASE3)	3
AT1G52340	ABA2 (ABA DEFICIENT 2)	1
AT5G15960	KIN1 cold and ABA inducible protein kin1	2
AT5G62490	ATHVA22B (Arabidopsis thaliana HVA22 homologue B)	1
AT1G74520	ATHVA22A (Arabidopsis thaliana HVA22 homologue A)	2
AT5G13200	GRAM domain-containing protein / ABA-responsive protein-related	3

Underexpressed

AGI	Description	Log2 Fold Change
AT3G43600	AAO2 (ALDEHYDE OXIDASE 2)	-1.227
AT3G19290	ABF4 (ABRE BINDING FACTOR 4)	-0.6
AT5G08350	GRAM domain-containing protein / ABA-responsive protein-related	-0.942
AT5G38760	similar to unknown protein (TAIR:AT5G53820.1)	-3.954

Figure S2. MAPMAN analysis of ABA-related genes with significant expression changes in pBISDCs transgenic plants with respect to WT plants.

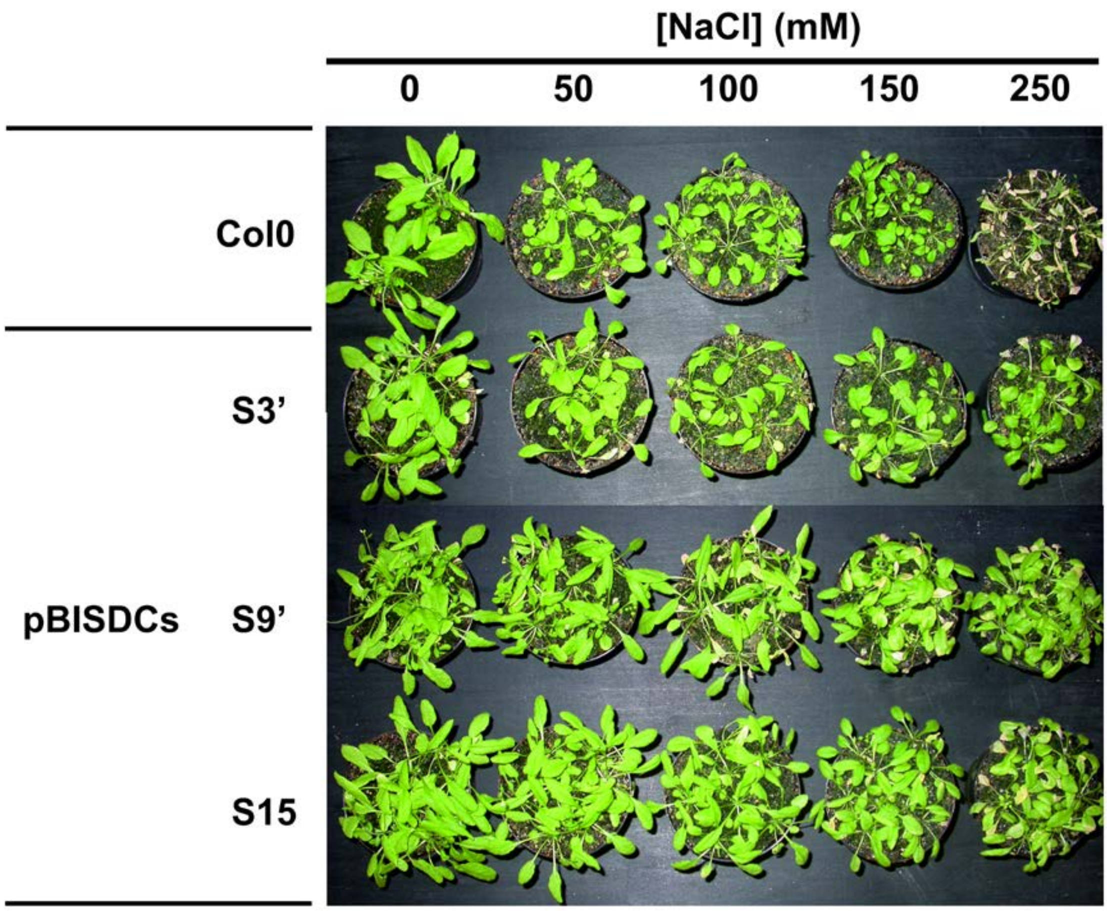

Figure S3. Appearance of Arabidopsis WT and pBISDCs transgenic plants overexpressing SAMDC1 (S3', S9 and S15) after 10 days of salt treatment. 3 week-old plants were watered with solution without (0) or with the supplementation of 50-250 mM NaCl. Pictures were taken after 10 days.

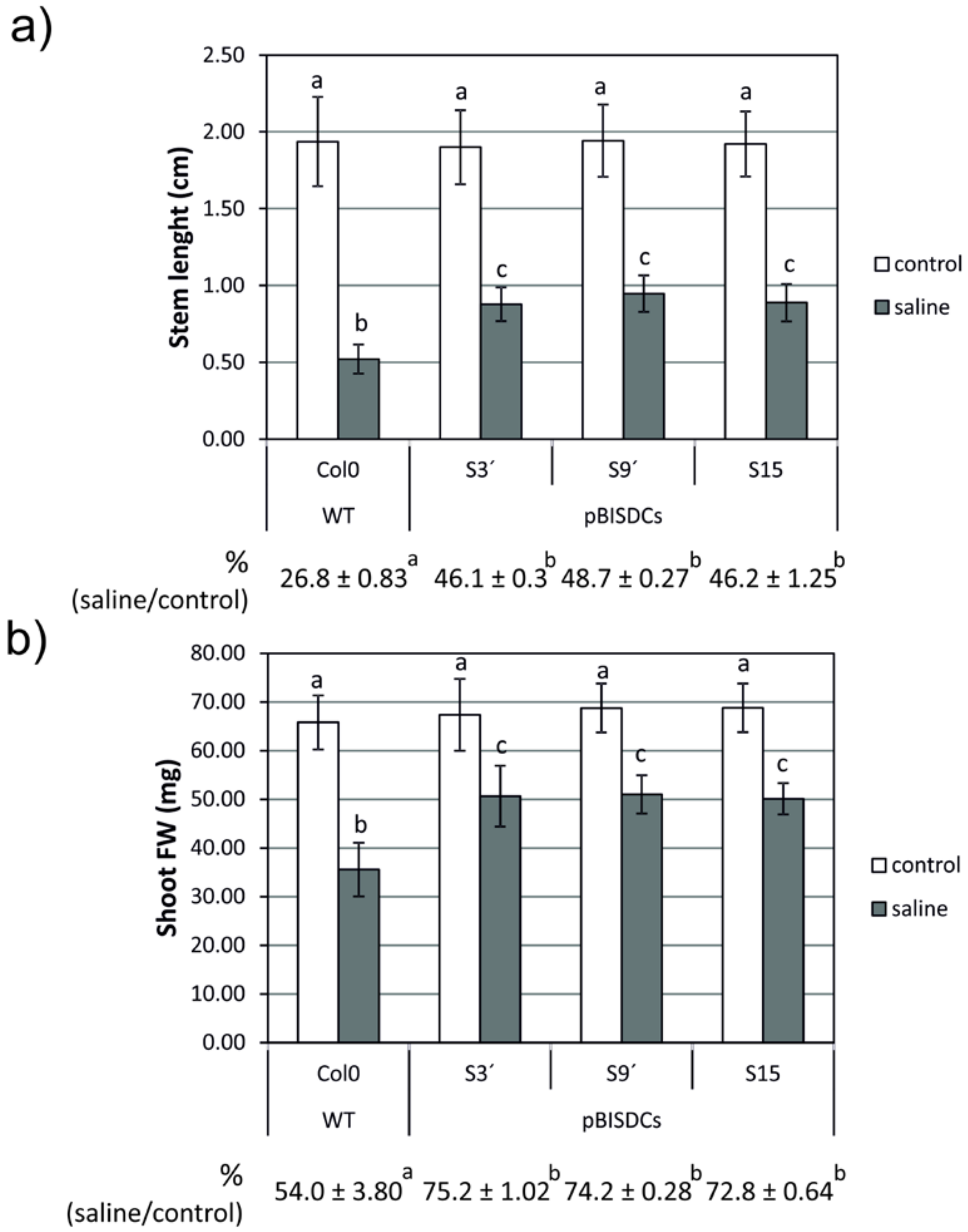

Figure S4. Saline stress recovery assay. Two week-old Arabidopsis WT and pBISDCs transgenic plants overexpressing SAMDC1 (S3', S9 and S15) plants were watered for 2 days with mild nutrient solution (control) or nutrient solution supplemented with 250mM NaCl (saline). After 12 days of recovery in control conditions, stem length and shoot fresh weight (FW) plant were measured. Graph show the mean of ten biological replicates ± standard deviation. Percentages were calculated with respect to the value of the parameter measured for each line grown in control conditions. Significant differences between plant lines are indicated with letters (ANOVA, Tukey HSD test, $p < 0.05$).

References:

Usadel, B., Nagel, A., Thimm, O., Redestig, H., Blaesing, O., Palacios-Rojas, N., et al. (2005). Extension of the visualization tool MapMan to allow statistical analysis of arrays, display of coresponding genes, and comparison with known responses. *Plant Physiology* 138**,** 1195 - 1204.
